# Patients’ experiences of promoting and hindering factors in daily physical activity with chronic obstructive pulmonary disease: a qualitative systematic review

**DOI:** 10.1186/s12890-026-04517-3

**Published:** 2026-07-17

**Authors:** Yongjia Zhang, Ying Xu, Yan Yang, Jingyou Sun

**Affiliations:** Department of Cardiology, West China Hospital, Sichuan University, No. 37, Guoxue Alley, Wuhou District, Chengdu City, Sichuan Province 610041 China

**Keywords:** Chronic Obstructive Pulmonary Disease, Barriers, Facilitators, Physical Activities of Daily Living, Qualitative Research, Systematic Review, Evidence-Based Nursing

## Abstract

**Background and Objectives:**

Physical activity (PA) is essential for maintaining function and quality of life in patients with chronic obstructive pulmonary disease (COPD). Yet, many return to sedentary behaviors after pulmonary rehabilitation. Identifying facilitators and barriers to daily activity is crucial for developing patient-centered interventions. However, qualitative evidence on patients’ experiences and perceptions remains limited.

**Methods:**

A qualitative systematic review was conducted following the Joanna Briggs Institute (JBI) Evidence Synthesis Manual. Twelve databases, including Cochrane Library, PubMed, Embase, CINAHL, and major Chinese databases, were searched up to December 5, 2023. Study quality was appraised using the JBI Checklist for Qualitative Research. Data were extracted with the JBI tool and synthesized using a meta-aggregative approach.

**Results:**

A total of 1,540 records were screened, and 11 studies were included. Forty findings were aggregated into 13 categories and synthesized into five themes: confronting symptom management and psychological challenges; lacking compliance, behavior change capacity, and autonomy in decision-making; stimulating motivations for engaging in activities; managing activity plans, respiratory aspects during activities, and activity safety; and optimizing family, peer, and medical support systems. Confidence in the synthesized findings ranged from low to high according to the ConQual assessment.

**Conclusions:**

COPD patients’ engagement in daily physical activity is influenced by a complex interplay of physical, psychological, behavioral, and social factors. These findings may inform the development of multidisciplinary, patient-centered strategies to support daily physical activity engagement among adults with COPD, including integrated symptom management, motivational support, individualized activity planning, safety monitoring, and strengthened social and professional support.

**Supplementary Information:**

The online version contains supplementary material available at 10.1186/s12890-026-04517-3.

## Introduction

Chronic obstructive pulmonary disease (COPD) is a major global public health concern [1], with a particularly high prevalence in China: 13.7% among individuals aged over 40 years and more than 27% among those over 60 years, causing disability in approximately 5–11 million people annually [2, 3]. Patients with COPD often experience reduced exercise capacity, impaired cardiopulmonary function, muscle mass loss, social isolation, and depressive symptoms, which are closely associated with dyspnea. Regular physical activity is considered a crucial intervention to improve prognosis and quality of life, encompassing structured programs such as pulmonary rehabilitation as well as daily physical activities (Physical Activity in Daily Life, PADL) [4]. Current clinical guidelines, including BMJ Best Practice, recommend that all COPD patients engage in regular physical activity [5]. However, in clinical practice, many patients experience exacerbated dyspnea during exercise [6], resulting in decreased adherence and self-efficacy, and a persistent tension between “need to exercise” and “fear of exercise”.

The Global Initiative for Chronic Obstructive Lung Disease (GOLD 2024) highlights that promoting and sustaining physical activity remains challenging [7]. Evidence from meta-analyses suggests that supervised home-based interventions following pulmonary rehabilitation can significantly improve functional capacity and health-related quality of life [8]. Although supervised and individualized home-based exercise programs have demonstrated benefits for patients with COPD, long-term adherence remains a major challenge. Recent work [9] has shown growing interest in home-based and remotely supported rehabilitation models after COPD exacerbation. However, sustaining daily physical activity remains challenging and is influenced by symptom burden, motivation, opportunities, and social/environmental support [10–13].

Quantitative studies can identify correlates of physical inactivity, but they are less able to explain how patients interpret breathlessness, negotiate fear and autonomy, or integrate activity into everyday routines [14–16]. Qualitative evidence is therefore essential for understanding the meanings, trade-offs, and contextual conditions underlying daily physical activity behavior. Recent qualitative studies have shown that symptoms such as dyspnea, fatigue, and sleep disturbances substantially limit daily activity and social participation among individuals with COPD [12]. However, evidence regarding how patients perceive, adapt to, and sustain daily physical activity across different life contexts remains fragmented. Understanding these lived experiences is important for developing patient-centered interventions that support long-term physical activity participation.

A recent review summarized the complex interplay among physical, psychological, social, and environmental factors influencing physical activity and sedentary behavior in COPD [13]. However, that review primarily focused on determinants and correlates of physical activity behavior rather than synthesizing patients’ subjective experiences of engaging in daily physical activity. Consequently, the ways in which individuals with COPD perceive, negotiate, and respond to barriers and facilitators in everyday life remain insufficiently synthesized. Therefore, this qualitative systematic review aimed to systematically aggregate evidence regarding patients’ experiences of daily physical activity and identify factors that facilitate or hinder sustained participation. By applying the JBI meta-aggregative approach and ConQual assessment, this review provides evidence-informed insights to support the development of patient-centered and multidisciplinary strategies for promoting physical activity in COPD.

## Methods

This review followed the JBI guidelines for systematic qualitative evidence synthesis [17] and adhered to the PRISMA guidelines for reporting [18]. The review protocol of the study was registered on the PROSPERO website with the ID CRD42024542681 without any modification.

### Information sources and search strategy

The lead author, in collaboration with expert librarians, selected search terms and keywords based on literature reviews. They refined and tested the strategy on PubMed and CNKI and applied it across various databases: Cochrane Library, Web of Science, PubMed, Embase, PsycINFO, CINAHL, Scopus, MEDLINE, SinoMed, CNKI, Wanfang Data, and VIP Databases. We also screened Google Scholar and ProQuest Global Theses and Dissertations to identify potentially relevant records and to check whether any peer-reviewed publications had been missed. However, grey literature itself, including dissertations, conference abstracts, reports, and unpublished studies, was excluded according to the eligibility criteria. The search spanned from inception to December 5, 2023, with detailed records maintained for each database, and references of relevant papers were checked. MeSH terms were combined with free terms, truncated where appropriate, and linked with “OR” operators within each group, combined with “AND.” Search strategies for each database can be found in Additional file 1 for transparency and reproducibility.

### Inclusion and exclusion criteria

Definition of physical activity (PA): In this review, physical activity was defined as any bodily movement produced by skeletal muscles that requires energy expenditure, including movement during leisure time, domestic activities, transport-related activities, and daily self-care activities [5]. In line with the focus of this review, we were primarily interested in PADL rather than structured exercise training alone.

Studies involving pulmonary rehabilitation (PR) were included only when they reported patients’ experiences, barriers, facilitators, or maintenance of PADL beyond supervised rehabilitation sessions.

Inclusion criteria: ① Participants (P) clinically diagnosed with chronic obstructive pulmonary disease (COPD), aged ≥ 18 years, conscious, and without cognitive impairments; ② Phenomena of interest (I) included attitudes, perspectives, or psychological experiences and perceptions during engagement in physical activities in daily living among individuals with COPD; ③ Context (Co) included settings such as hospitals, rehabilitation centers, communities, or homes where physical activities occur; ④ Study design (S) encompassed original qualitative research designs, including phenomenology, grounded theory, ethnography, action research, and feminist studies, among others.

Exclusion criteria: ① Non-English or non-Chinese publications; ② Unavailable full texts, incomplete data, or duplicate publications; ③ Quantitative-only studies; ④ Intervention efficacy studies without qualitative data; ⑤ Mixed-methods studies in which qualitative findings could not be separately extracted; ⑥ Studies focusing solely on structured exercise/PR attendance without daily physical activity experience; ⑦Grey literature without sufficient methodological information or extractable qualitative findings was excluded.

### Selection process

Based on predefined inclusion and exclusion criteria, two reviewers (YZ and YY), trained in evidence-based nursing, and a third reviewer (YX) conducted a two-stage article screening process following the PRISMA guidelines [18]. In the first stage, titles and abstracts were independently reviewed, and citations deemed uncertain were forwarded for full-text assessment. In the second stage, full texts were thoroughly evaluated, and inclusion decisions were made after collective discussion. Data extraction and cross-checking were independently performed by two reviewers (YZ and YY) using NoteExpress, with any discrepancies resolved through consultation with the third reviewer (YX). For studies with incomplete data, the authors were contacted; those who did not respond within two weeks were excluded.

### Assessment of methodological quality

Using the JBI Critical Appraisal Checklist for Qualitative Research, two reviewers (YZ and YY) independently evaluated quality. This tool, guided by the JBI, ensures evaluation consistency and reliability through systematic standardization. It assesses various qualitative research aspects comprehensively, identifying biases and limitations. Discrepancies in assessments were resolved through discussion with the third reviewer (YX). Evaluation criteria included 10 items rated as “yes,” “no,” “unclear,” and occasionally, “not applicable.” Studies meeting all criteria were classified as Level A; partially meeting, as Level B; and those not meeting, as Level C. Studies rated A and B were included to enhance credibility, excluding C-rated studies.

### Data extraction

Using the JBI Qualitative Data Extraction Tool, two reviewers (YZ and YY) independently extracted and cross-checked data from the included literature, validated by the third reviewer (YX). Before full extraction, two randomly selected studies underwent trial runs and adjustments. The final data extraction form is available in Additional file 1. Subsequently, the authors evaluated the credibility of extracted results, categorizing them as unequivocal, credible, or unsupported based on consistency with the provided descriptions [19].

### Data synthesis

The study employed the data synthesis method recommended by the JBI guidelines for systematic qualitative evidence synthesis, consisting of three steps, in a meta-aggregative review to collect research findings and further integrate and summarize them based on their implications. After establishing a thorough understanding of qualitative research philosophy and methodology, two reviewers (YZ and YY) initially extracted all study findings from the included papers along with illustrations. Categories were then developed for similar findings, ensuring each category included findings from at least two studies. Syntheses were conducted for findings across multiple categories. Through iterative reading and textual analysis, results reflecting similar concepts based on participants’ original experiences were re-summarized into new categories. These findings were continuously discussed in team meetings until a consensus was reached among all authors, consolidating new categories into integrated findings. Each synthesis was assigned a ConQual score to assess result dependability or credibility [20].

Data synthesis followed JBI guidance for qualitative evidence synthesis and meta-aggregation [17, 19]. The ConQual approach [20] was used to assess confidence in each synthesized finding after meta-aggregation. This additional assessment was necessary because synthesized qualitative findings may vary in methodological dependability and credibility, even when they are generated through the same synthesis process. ConQual, therefore, provides readers with a transparent judgment about the extent to which each synthesized finding can be relied upon for practice and future research.

Each extracted finding was assigned a credibility level according to JBI guidance. An unequivocal finding was defined as a finding accompanied by an illustration that was beyond reasonable doubt and, therefore, not open to challenge. A credible finding referred to a finding supported by an illustration but with a less clear association, and thus open to challenge. An unsupported finding referred to a finding not supported by the data. Credible findings were not excluded; rather, they were retained when they were conceptually relevant and supported by at least some participant data, but their presence was considered when downgrading the credibility component of the corresponding ConQual score [20].

### Researcher reflexivity

Reflexivity was considered throughout data extraction and synthesis. The review team included researchers with backgrounds in evidence-based nursing and clinical care of patients with cardiopulmonary conditions. These backgrounds may have sensitized the team to issues of symptom management, adherence, and patient education. To reduce the influence of pre-existing assumptions, two reviewers independently extracted and coded findings, retained participants’ original illustrations alongside extracted findings, discussed discrepant interpretations in team meetings, and avoided introducing themes that were not supported by primary data.

## Results

### Study inclusion

**Fig. 1:**
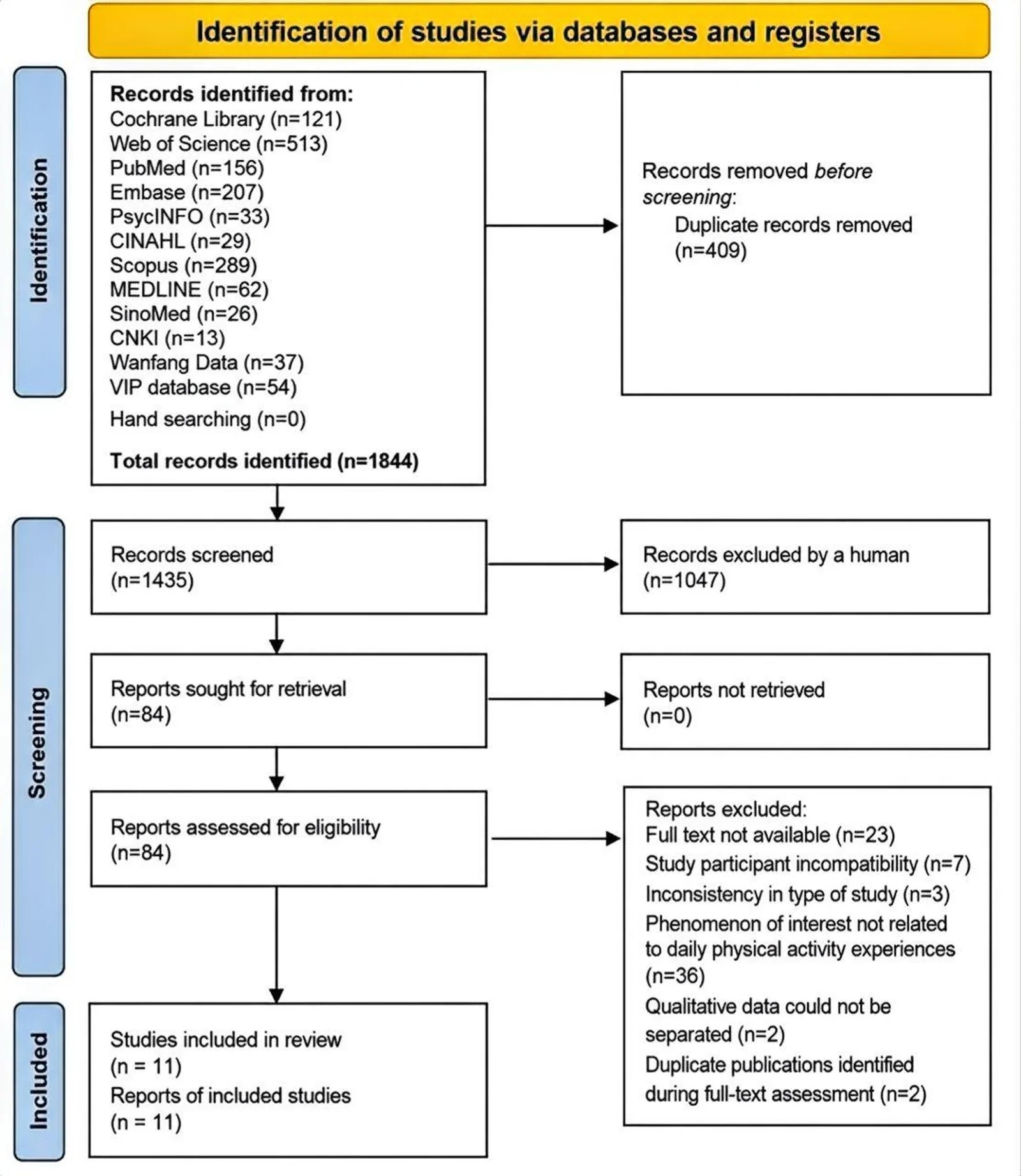
PRISMA flowchart

Following the retrieval strategy, a total of 1540 articles were initially identified. After removing duplicates, 1131 articles remained. Initial screening of titles and abstracts yielded 84 articles, of which 11 were selected after full-text review. The literature selection process is illustrated in Fig. 1.

### Methodological quality

Table 1 summarizes the critical appraisal results of the included studies (11 in total). Of these, three studies [21–23] were graded as A-level, meeting all 10 JBI criteria with a “Yes” rating. The remaining eight studies [24–31] were classified as B-level, each failing one or more items.

The most frequently unmet domain was item 6 (locating the researcher culturally or theoretically), which was marked as “Unclear” in four studies and “No” in two studies. Item 7 (consideration of the researcher’s influence on the research process) was also occasionally rated “Unclear. ” In addition, isolated “No” or “Unclear” ratings were observed in items 4 and 9 in a small number of studies.

**Table 1:** Critical appraisal of eligible studies

Study	Q1	Q2	Q3	Q4	Q5	Q6	Q7	Q8	Q9	Q10	Quality level
Chauvin et al., [21]	Y	Y	Y	Y	Y	Y	Y	Y	Y	Y	A
Chang et al., [22]	Y	Y	Y	Y	Y	Y	Y	Y	Y	Y	A
Kaptain et al., [24]	Y	Y	Y	Y	Y	U	Y	Y	Y	Y	B
Michalovic, [25]	Y	Y	Y	U	Y	Y	Y	Y	Y	Y	B
Luo et al., [26]	Y	Y	Y	Y	Y	U	Y	Y	Y	Y	B
Lahham et al., [27]	Y	Y	Y	Y	Y	U	Y	Y	Y	Y	B
Kosteli et al., [28]	Y	Y	Y	Y	Y	U	Y	Y	Y	Y	B
Thorpe et al., [29]	Y	Y	Y	Y	Y	N	U	Y	Y	Y	B
Hogg et al., [23]	Y	Y	Y	Y	Y	Y	Y	Y	Y	Y	A
Jeng et al., [30]	Y	Y	Y	Y	Y	N	Y	Y	U	Y	B
Leidy et al., [31]	Y	Y	Y	Y	Y	U	Y	Y	U	Y	

*Y *Yes, *N *No, *U *Unclear, *N/A *not applicable. Additional file 1 contains critical appraisal questions

### Characteristics of included studies

A total of 11 qualitative studies published between 1996 and 2022 were included in this review. The studies were conducted across a wide range of countries, including Canada, Singapore, Denmark, China, Australia, the United Kingdom, and the United States. All investigations adopted qualitative approaches, most commonly semi-structured or focus group interviews.

Participants were exclusively individuals living with COPD, ranging from newly diagnosed patients to those with moderate or severe disease, as well as individuals who had recently experienced acute exacerbations or completed pulmonary rehabilitation programs. Sample sizes varied from 6 to 28 participants, with ages spanning from 50 to 89 years.

The phenomena of interest primarily centered on experiences of PA, barriers and facilitators to participation, perceptions of pulmonary rehabilitation, and strategies for managing daily life activities. Data collection was conducted in diverse settings, including patients’ homes, outpatient clinics, community hospitals, and universities, with interviews often taking place after discharge, rehabilitation completion, or outpatient visits. Further details on study characteristics are presented in Table 2.

**Table 2:** Study characteristics

Author	Country	Year	Participants	Sample (Female *n*)	Age range/ Average (years)	Phenomena of interest	Methodology and method	Interview start time and setting
Chauvin et al. [21]	Canada	2022	Elderly COPD patients	15(11)	74.6 ± 7.5	Experiences and facilitators/ barriers of participating in family fall prevention exercise programs	Explanatory qualitative study; semi-structured interviews	After completing at least three months of the home fall prevention program, telephone interviews were conducted
Chang et al. [22]	Singapore	2022	COPD patients admitted for acute exacerbation	6(0)	72 ± 5	Factors influencing patients’ adoption of sedentary behaviors and PA patterns following acute exacerbations	Phenomenological research; semi-structured interviews	Seven to fourteen days after discharge, participants were interviewed at their homes
Kaptain et al. [24]	Denmark	2022	COPD patients enrolled in PR and telemedicine programs	8(4)	73.5 ± 7.8	Experiences, execution, and management of daily life activities	Qualitative study; semi-structured interviews	Participants’ preferred time was selected, typically at their homes
Michalovic [25]	Canada	2021	COPD patients at various stages of the disease	14(5)	71.4 ± 7.4	Past PA experiences, acceptability of peer-supported and daily engagement PA interventions	Descriptive qualitative study; focus group interviews	Each focus group was conducted at the researcher’s university after recruiting 4–5 people
Luo et al. [26]	China	2020	COPD patients in stable condition	18(3)	61.9 ± 6.2	Understanding experiences, facilitators, and barriers to PA	Phenomenological research; semi-structured interviews, observational methods	During patients’ outpatient follow-up visits, interviews were conducted in the outpatient waiting area
Lahham et al. [27]	Australia	2018	COPD patients with a smoking history of 10 pack-years or more	13(10)	66.4 ± 7.4	Perceptions of home-based PR	Qualitative study; semi-structured interviews	After completing an eight-week home PR program, interviews were conducted face-to-face or via telephone
Kosteli et al. [28]	United Kingdom	2017	COPD patients who are newly diagnosed, have participated in PR, are employed, and are at different stages of the disease	26(11)	50–89	Factors encouraging and inhibiting engagement in PA in primary care	Qualitative study; focus group interviews	Each focus group was conducted at the researcher’s university after recruiting 5–8 people
Thorpe et al. [29]	Australia	2014	COPD patients admitted for acute exacerbation	28(6)	71.86	Barriers and facilitators to post-hospitalization PA participation	Descriptive qualitative study; semi-structured interviews	Two months after hospital admission, telephone interviews were conducted
Hogg et al. [23]	United Kingdom	2012	COPD patients who completed an 8-week outpatient PR program within the past two years	16(7)	69 ± 10.5	Views on maintaining an active lifestyle post-PR	Grounded theory; focus group interviews	Upon receiving nine affirmative responses from each group, interviews were conducted at the community hospital
Jeng et al. [30]	China	2002	COPD patients with limited PA	9(2)	65–80	Experiences of daily activities within two weeks after discharge	Qualitative study; semi-structured interviews	Two weeks after discharge, participants were interviewed at their homes
Leidy et al. [31]	United States	1996	COPD patients with moderate to severe disease	12(6)	66.8	Current and past participation in activities, influencing factors, and adjustment strategies	Qualitative study; unstructured interviews	Interviews were conducted in the outpatient office before and after outpatient visits

*COPD *Chronic obstructive pulmonary disease, *PR *Pulmonary rehabilitation, *PA *Physical activity

### Findings of the review

Through iterative reading and analysis of the 11 selected articles, 40 research findings were extracted. Among these, 37 were unequivocal, 3 were credible, and none were unsupported. These findings were then categorized into 13 new categories, contributing to the synthesis of 5 integrated research outcomes. Table 3 encapsulates the findings, categories, and synthesized findings. Illustrations can be located in Additional file 1.

**Table 3:** Findings, categories, and synthesized findings

Synthesized finding	Category	Findings	Credibility level
S1: Confronting symptom management and psychological challenges	C1: Facing with fluctuations in symptoms and post-activity discomfort	F1: Variability and unpredictability of symptoms	U
		F2: Affected by acute deterioration of the condition	U
		F3: Experienced difficulty breathing after activities	U
		F4: Experienced fatigue after activities	U
	C2: Encountering psychological and emotional challenges	F5: Low self-efficacy in community activities	U
		F6: Concerns about breathing difficulties and pain	U
		F7: Fear of becoming an additional burden to family members	U
S2: Lacking compliance, behavior change capacity, and autonomy in decision-making	C3: Experiencing non-adherence and individual differences	F8: Poor adherence to exercise recommendations	U
		F9: Varying levels of personal physical health	U
	C4: Lacking the ability to translate intentions into actions	F10: Aging limits on activity capability	U
		F11: Economic burden of carrying oxygen equipment	U
		F12: Seasonal weather patterns reduce outdoor activity ability	C
	C5: Experiencing an imbalance between social support and autonomous decision-making	F13: Overprotection by family members impedes patient activity	U
		F14: Resistance to dependency	U
S3: Stimulating motivations for engaging in activities	C6: Striving for personal goals and psychological rewards	F15: Alignment of activity projects with personal goals	U
		F16: Achievement of personal goals	C
		F17: Social interaction through activities	U
		F18: Preparation for upcoming events	U
		F19: Seeking satisfaction through activities	U
	C7: Self-motivating and receiving intuitive feedback	F20: Self-motivation through commitment, determination, and guilt	U
		F21: Visual motivation through testing and feedback	U
S4: Managing activity plans, respiratory aspects during activities, and activity safety	C8: Coordinating activities and managing respiratory difficulties	F22: Building confidence in activity through controlled breathing	U
		F23: Awareness of pollen and dust in activity environments	U
		F24: Adapting to personal activity capabilities	U
		F25: Increasing rest time and simplifying activities	U
	C9: Adapting daily activity schedules flexibly	F26: Incorporating activities into daily life and setting goals	U
		F27: Problem-solving and activity planning	U
		F28: Adjusting based on health conditions	U
		F29: Proceeding within one’s capabilities	U
	C10: Strengthening self-management of activity safety	F30: Gradual progression	U
		F31: Selecting appropriate assistive tools	U
S5: Optimizing family, peer, and medical support systems	C11: Providing personalized family support	F32: Special care from family members	U
		F33: Understanding and encouragement from spouse	U
	C12: Enhancing peer support networks	F34: Peer support enhances activity capabilities	U
		F35: Establishing connections within peer groups	U
		F36: Lack of community activity environments	U
	C13: Expanding the content and models of medical guidance	F37: Disappointing experience with inpatient physical therapy	C
		F38: Encouragement and professional guidance from attending physicians	U
		F39: Home visits and follow-ups by physical therapists	U
		F40: Participation in activity programs via online platforms	U

*U* unequivocal, *C *credible. No unsupported findings were included

#### Synthesized finding 1: confronting symptom management and psychological challenges

Patients commonly reported the unpredictability and discomfort of symptoms, including post-activity dyspnea, fatigue, and acute exacerbations, alongside low self-efficacy in community activities, concern about breathing difficulties, and fear of becoming a burden to family members [21, 24, 25, 29]. The interplay between physical symptom burden and psychological stress formed an inseparable whole, representing a primary obstacle and the “first barrier” to activity engagement.

#### Synthesized finding 2: lacking compliance, behavior change capacity, and autonomy in decision-making

Patients exhibited limitations in adherence, behavioral change capacity, and decision-making autonomy. Individual health status differences and age-related decline constrained physical capability, while economic and environmental factors, such as the cost of carrying oxygen equipment and seasonal weather changes, posed objective barriers. Overprotective family behaviors further restricted autonomy and sometimes elicited “resistance to dependence,” highlighting the delicate balance between social support and personal agency [24–29]. Collectively, these factors contributed to a gap between intention and sustained activity behavior.

#### Synthesized finding 3: stimulating motivations for engaging in activities

Motivation to engage in activity was driven by multiple internal and external factors, including alignment of activities with personal goals, attainment of a sense of accomplishment, participation in social interactions, and preparation for future events. Self-regulation strategies, such as commitment, determination, and feelings of guilt, as well as visual feedback from monitoring or testing, further reinforced sustained participation [21, 22, 28, 30].

#### Synthesized finding 4: managing activity plans, respiratory aspects during activities, and activity safety

Patients adopted diverse strategies to manage activity planning, respiration, and safety, including controlled breathing, increasing rest periods, simplifying tasks, adapting activities to personal capacity, and gradual progression. Awareness of environmental factors, such as pollen and dust, and integrating activities into daily routines with goal-setting, planning, and problem-solving, facilitated safe, individualized, and sustainable participation [21, 22, 26].

#### Synthesized finding 5: optimizing family, peer, and medical support systems

Family, peer, and healthcare support systems played a critical role in facilitating engagement. Family provided personalized care beyond overprotection and spousal encouragement; peer support enhanced activity capability, offered modeling, and fostered a sense of belonging; and healthcare professionals extended traditional guidance through encouragement, follow-up, home visits, and online activity programs, improving expertise, continuity, and accessibility [21–23, 26, 27, 30]. These multi-level supports collectively promoted participation in PA among COPD patients.

### ConQual summary

Table 4 provides the ConQual summary derived from the synthesised findings, presenting the dependability, credibility, and overall ConQual scores across different aspects of daily physical activity among adults with COPD. Dependability was generally rated as moderate to high, reflecting a consistent methodological quality across included studies. Credibility, however, varied, with some findings being downgraded. Consequently, the overall ConQual scores ranged from low to high. Stronger evidence emerged in relation to confronting symptom management and psychological challenges, where both dependability and credibility supported a high ConQual rating. In contrast, findings concerning compliance, behavior change, and autonomy in decision-making were underpinned by only low-level evidence. Areas such as motivation for engagement, management of activity planning and safety, and the role of support systems achieved moderate confidence levels. Taken together, while the evidence base demonstrates methodological soundness, the strength of certainty remains uneven, highlighting the need for further high-quality research to reinforce the evidence in domains of self-management, behavioral adherence, and supportive strategies

**Table 4:** Summary of findings table

Synthesized Finding	Dependability	Credibility	ConQual Score
Confronting symptom management and psychological challenges	High (scored 5/5 for the 5 criteria in 2 studies, 4/5 in 4 studies)	Remains unchanged	High
Lacking compliance, behavior change capacity, and autonomy in decision-making	Moderate (scored 5/5 for the 5 criteria in 2 studies, 4/5 in 3 studies and 3/5 in 1 study)	Downgraded one level	Low
Stimulating motivations for engaging in activities	High (scored 5/5 for the 5 criteria in 3 studies, 4/5 in 3 studies)	Downgraded one level	Moderate
Managing activity plans, respiratory aspects during activities, and activity safety	Moderate (scored 5/5 for the 5 criteria in 3 studies, 4/5 in 4 studies and 3/5 in 1 study)	Remains unchanged	Moderate
Optimizing family, peer, and medical support systems	High (scored 5/5 for the 5 criteria in 2 studies, 4/5 in 4 studies)	Downgraded one level	

All synthesized findings were derived from qualitative studies

## Discussion

Consistent with the conceptual framework proposed by Tang et al., our findings suggest that participation in daily physical activity is influenced by the dynamic interaction of symptom burden, psychological factors, behavioral regulation, and social support. This review identified five synthesized findings regarding barriers and facilitators of daily physical activity among adults with COPD. Specifically, symptom burden and psychological challenges emerged as major barriers to physical activity participation, whereas motivation, self-management strategies, and supportive social environments acted as important facilitators of sustained engagement. Together, these findings highlight the complex interaction between physical, psychological, behavioral, and social factors that shape daily physical activity experiences in COPD. These dimensions do not exist in isolation but constitute an interrelated system: challenges in one domain often exacerbate difficulties in another, whereas facilitators can create a positive feedback loop that supports the maintenance of health-promoting behaviors. These findings suggest that interventions aimed at supporting physical activity participation in individuals with COPD may benefit from a multidimensional approach that integrates symptom management, behavioral support, self-management education, and social support mechanisms [32, 33].

Confronting symptom management and psychological challenges remains a primary barrier to physical activity participation. Patients commonly experience exertional dyspnea, fatigue, and the unpredictability of symptoms, alongside anxiety, depression, and fear of becoming a family burden. Zeng et al. indicated that dyspnea and fatigue significantly impede adherence to home-based pulmonary rehabilitation, with associated psychological stress further reducing activity engagement [34]. Similarly, Wang et al. highlighted that psychological distress and inadequate coping strategies are closely linked to activity avoidance behaviors [35]. Breathlessness and fatigue may contribute to activity avoidance behaviors, as individuals often restrict physical exertion to minimize symptom exacerbation. Prolonged reductions in activity can result in physical deconditioning, diminished exercise capacity, and further symptom burden, thereby perpetuating a cycle of inactivity. Almonacid et al. further emphasized that patients with COPD experience continuous support needs throughout their disease trajectory, including emotional and informational support, which directly affect their capacity to manage symptoms and maintain activity levels [36]. Taken together, these findings indicate that symptom burden and psychological distress are closely intertwined and may need to be addressed concurrently within clinical practice. Future clinical interventions may therefore need to integrate symptom management with psychological support [37], including cognitive-behavioral strategies, emotion regulation, and anxiety management, to holistically enhance patients’ activity capacity and quality of life.

Lack of adherence, behavior change capacity, and autonomy in decision-making significantly affect activity maintenance. Alamer et al. found that health literacy and unequal access to resources impact adherence to COPD interventions among ethnic minority populations [38]. Raghavan et al. further indicated that overreliance on healthcare guidance limits patient autonomy, reducing intrinsic motivation for behavior change [39]. Wu et al. highlighted that variations in health information literacy among COPD patients are closely associated with differences in self-management capacity and quality of life, indicating that patients with limited literacy face greater difficulties in independent decision-making and activity adaptation [40]. Consistent with these studies, our findings suggest that patients often lack self-management awareness and decision-making capacity, impeding activity adaptation. The synthesis indicates the potential value of multidisciplinary strategies that emphasize self-management education, behavioral skills training, and shared decision-making. Such strategies may help patients co-develop personalized activity plans and support sustained participation.

Stimulating motivation for engaging in activities is essential for sustaining participation among individuals with COPD. Chien et al. demonstrated that digital health technologies incorporating monitoring and feedback enhanced patients’ self-efficacy and willingness to engage in PA [41]. Similarly, Iyer et al. showed that individualized goal setting, staged feedback, and motivational strategies significantly improved adherence to daily activities [42]. These findings are consistent with Self-Determination Theory [43], which proposes that autonomy, competence, and relatedness are fundamental psychological needs underpinning sustained health behavior. Opportunities for goal attainment, positive feedback, and supportive relationships may therefore strengthen intrinsic motivation and facilitate long-term engagement in PA. Consistent with this perspective, participants in the included studies described greater motivation when activity goals reflected their individual needs, health status, and life priorities, such as maintaining valued roles, social participation, or personally meaningful routines. These findings suggest that strategies to support sustained engagement should be individualized and responsive to patients’ changing circumstances. Healthcare interventions may incorporate individualized goal setting, real-time feedback, and supportive coaching approaches to strengthen self-efficacy and promote long-term participation in daily physical activity.

Activity plan management, respiratory control, and safety are critical for ensuring effective participation. Debeij et al. highlighted that wearable monitoring devices provide real-time data to help patients adjust exercise intensity and reduce fatigue or adverse events [44]. Salmi et al. emphasized individualized activity planning, breathing technique training, and fatigue management as essential for safety and adherence [45]. Consistent with these findings, our synthesis suggests that insufficient guidance may make it more difficult for patients to adjust activity intensity, manage symptoms, and maintain confidence during activity. These findings suggest that future healthcare interventions may need to incorporate comprehensive assessments of physical and respiratory capacity, tailored activity planning, breathing guidance, and safety monitoring to promote safe and effective physical activity.

Optimizing family, peer, and medical support systems acts as an external facilitator. Jehloh et al. found that emotional and practical support from family members and nurses during discharge transitions enhanced patients’ self-efficacy and adherence [46]. Alamer et al. noted that culturally sensitive social support is especially crucial for minority patients [38]. Although the present review did not conduct a cross-cultural comparison, previous research has suggested that the form and accessibility of social support may differ across population groups. Almonacid et al. also highlighted the importance of coordinated support across the patient journey, emphasizing that multidimensional assistance from healthcare providers, family, and peers strengthens patients’ confidence and consistency in activity participation [36].

Our findings suggest that multidimensional support networks, including family assistance, peer encouragement, and multidisciplinary medical guidance, may help create a safer and more sustainable environment for activity participation. Clinical practice may benefit from integrating family education, peer support groups, and interprofessional collaboration to systematically enhance patients’ engagement in daily physical activity.

## Limitations

This review is constrained by several limitations.

First, although the review focused on physical activity in daily life (PADL), several included studies were conducted in pulmonary rehabilitation or post-rehabilitation contexts. These studies were included only when they reported experiences related to daily physical activity beyond supervised rehabilitation sessions; nevertheless, some findings may still partially reflect rehabilitation-related experiences.

Second, the included studies did not apply fully consistent distinctions among physical activity, exercise, and pulmonary rehabilitation. Despite the predefined eligibility criteria, some conceptual overlap may therefore remain across the synthesized findings.

Third, only studies published in Chinese or English were included, potentially excluding relevant evidence from other linguistic and cultural contexts. Furthermore, although the included studies were conducted across several countries, this review was not designed to undertake a formal cross-cultural comparison. Therefore, findings related to family, peer, and professional support should not be interpreted as universally transferable across all sociocultural settings.

Fourth, methodological heterogeneity across the included studies may have affected comparability and synthesis consistency. The studies employed a range of qualitative data collection methods, including semi-structured interviews, focus group discussions, unstructured interviews, and observation. In addition, variation in participant characteristics, such as gender, age, disease severity, and cultural background, may limit the applicability of the findings to specific populations.

Finally, the review included 165 participants, most of whom were older than 60 years, with relatively limited representation of women and younger individuals with COPD. Future research should therefore explore the experiences of female and younger patients in greater depth. Moreover, confidence in the synthesized findings ranged from low to high according to the ConQual approach, indicating that some implications for practice should be interpreted with caution.

## Conclusions

This review synthesized qualitative evidence on adults’ experiences of promoting and hindering factors in daily physical activity with COPD. The findings indicate that daily physical activity is shaped by symptom burden, psychological responses, behavioral regulation, activity planning, safety concerns, and family, peer, and professional support. These findings may inform the development of multidisciplinary, patient-centred strategies to support daily physical activity engagement. Future research should further examine how these synthesized findings can be translated into interventions and evaluated across diverse clinical and sociocultural contexts.

## Supplementary Information

Supplementary Material 1.

## Data Availability

All data generated or analysed during this study are included in this published article and its supplementary information files.
