# Supplementary material for: Patients’ experiences of promoting and hindering factors in daily physical activity with chronic obstructive pulmonary disease: a qualitative systematic review
Source: BMC Pulm Med. 2026 Jul 17;26:367. doi: 10.1186/s12890-026-04517-3 (PMC13491779; doi:10.1186/s12890-026-04517-3)
Supplement: Supplementary file 1 [file 12890_2026_4517_MOESM1_ESM.docx]

**Appendix 1: Search strategy**

|  | **Population** | **Interest of phenomena** | | **Study design** |
| --- | --- | --- | --- | --- |
| MeSH Terms | pulmonary disease, chronic obstructive | exercise/exercise therapy/sports | - | qualitative research/grounded theory/narration/focus groups |
| Free text terms | chronic obstructive lung disease/chronic obstructive pulmonary disease/COPD | physical activity/physical exercise/physical behavior/rehabilitation/fitness/exergaming/gymnastics/training/ swimming/walking/stair climbing/Tai Ji | experience/  feeling/  attitude/  expectation/  desire/emotion | document analysis/hermeneutics/qualitative study/content analysis/discourse analysis/ethnographies/focus interview/semistructured interview/phenomenology/thematic analysis |
| Chinese search terms | 慢性阻塞性肺疾病/慢阻肺 | 体力活动/身体活动/体育活动/身体行为/运动/康复/训练/锻炼/家务/职业活动/工作活动/交通出行/爬楼梯/步行/骑车/散步/太极拳/八段锦/游泳 | 体验/感受/心理/态度/看法/经历/想法 | 定性研究/质性研究/内容分析/民族志/焦点访谈/半结构式访谈/扎根理论/叙事/现象学/主题分析 |

**Search Strategy**

**Database: Cochrane Library**	Date of Search: 5/12/2023	No. of Results:121

**Database: Web of Science**	Date of Search: 5/12/2023	No. of Results: 513

**Database: PubMed**	Date of Search: 5/12/2023	No. of Results:156

**Database: Embase**	Date of Search: 5/12/2023	No. of Results:207

**Database: PsycINFO**	Date of Search: 5/12/2023	No. of Results:33

**Database: CINAHL**	Date of Search: 5/12/2023	No. of Results:29

**Database: Scopus**	Date of Search: 5/12/2023	No. of Results:289

**Database: MEDLINE**	Date of Search: 5/12/2023	No. of Results:62

**Database: SinoMed**	Date of Search: 5/12/2023	No. of Results:26

**Database: CNKI**	Date of Search: 5/12/2023	No. of Results:13

**Database: Wanfang Data**	Date of Search: 5/12/2023	No. of Results:37

**Database: VIP Database**	Date of Search: 5/12/2023	No. of Results:54

**Appendix 2: Critical appraisal checklist**

**Appendix 3: Data extraction instrument**

**Appendix 4: List of excluded studies**

| **No.** | **Year of publication** | **First author** | **Title** | | **Remarks** |
| --- | --- | --- | --- | --- | --- |
| 1 | 2022 | Chang | | Understanding perspectives and choices for sedentary behaviour and physical activity in older adults' post-acute exacerbation of chronic obstructive pulmonary disease | Included |
| 2 | 2022 | Kaptain | | Juggling the management of everyday life activities in persons living with chronic obstructive pulmonary disease |  |
| 3 | 2022 | Chauvin | | Experiences of a home-based fall prevention exercise program among older adults with chronic lung disease |  |
| 4 | 2021 | Michalovic | | Participation in Daily and Social Activities in Individuals Living with COPD: The Role of Peer Support and Physical Activity |  |
| 5 | 2020 | Luo | | Daily Physical Activity in Patients Living with COPD: a Qualitative Study |  |
| 6 | 2018 | Lahham | | Home-based pulmonary rehabilitation for people with COPD: A qualitative study reporting the patient perspective |  |
| 7 | 2017 | Kosteli | | Barriers and enablers of physical activity engagement for patients with COPD in primary care |  |
| 8 | 2014 | Thorpe | | Barriers to and enablers of physical activity in patients with COPD following a hospital admission: a qualitative study |  |
| 9 | 2012 | Hogg | | People with COPD perceive ongoing, structured and socially supportive exercise opportunities to be important for maintaining an active lifestyle following pulmonary rehabilitation: a qualitative study |  |
| 10 | 2002 | Jeng | | Experiences of Daily Activities Within Two Weeks After Hospital Discharge Among Taiwanese Elderly Patients With Chronic Obstructive Pulmonary Disease |  |
| 11 | 1996 | Leidy | | Functional Performance in People with Chronic Obstructive Pulmonary Disease: A Qualitative Analysis |  |
| 12 | 2023 | Rebelo | | Effectiveness of PersonalIsed CommUnity-based Physical activities for people with chronic obstructive pulmonary disease (PICk UP) | Full text not available |
| 13 | 2021 | Lewis | | Singing for Lung Health’ group participation after pulmonary rehabilitation completion. A feasibility study |  |
| 14 | 2020 | Benzo | | Randomized Study of Home-Based Rehabilitation with Health Coaching in Chronic Obstructive Pulmonary Disease |  |
| 15 | 2019 | Norweg | | Respiratory patient perspectives on exercising with music: A qualitative study |  |
| 16 | 2019 | Thomas | | Home-Based Rehabilitation for COPD: A Qualitative Analysis of a Large Randomized Study |  |
| 17 | 2019 | Santos | | Enablers, barriers, needs and preferences of physical activity in patients with COPD: Does pulmonary rehabilitation play a role? |  |
| 18 | 2019 | Simonÿ | | Breathing through a troubled life - a phenomenological-hermeneutic study of chronic obstructive pulmonary disease patients' lived experiences during the course of pulmonary rehabilitation |  |
| 19 | 2018 | Pilsworth | | Group-based social identity intervention during pulmonary rehabilitation improves COPD patients experience and promotes compliance with exercise programme |  |
| 20 | 2017 | MacBean | | Dance as a rehabilitative strategy for patients with COPD |  |
| 21 | 2016 | 陈奕 | | 慢性阻塞性肺疾病患者实施居家力量训练感受的质性研究 |  |
| 22 | 2016 | 陈奕 | | 坚持康复锻炼五年以上的慢性阻塞性肺疾病患者的生活体验 |  |
| 23 | 2016 | Zakrisson | | Using a mobile app to motivate exercise in persons with COPD: A mixed method study |  |
| 24 | 2016 | Chen | | Perceives and experiences of participating in a combined biofeedback with pulmonary rehabilitative exercise training program for patients with chronic obstructive pulmonary disease |  |
| 25 | 2016 | Lima | | Factors influencing participation and adherence to a pulmonary rehabilitation program in patients with COPD: A qualitative study |  |
| 26 | 2016 | Soerensen | | Experiences of training-adherence in a 12 weeks home-based IMT program for individuals with COPD |  |
| 27 | 2014 | Marques | | Pulmonary Rehabilitation Programs In COPD: The Patients' Perspective |  |
| 28 | 2012 | Lewis | | Chronic obstructive pulmonary disease (COPD) patient experiences of pulmonary rehabilitation (PR): A longitudinal qualitative UK study |  |
| 29 | 2011 | De Jong | | Experiences of physical activity are similar among European COPD patients |  |
| 30 | 2011 | Sundfor | | Daily activity habits, energy conservation methods and activity training for patients with COPD. A qualitative study |  |
| 31 | 2011 | Dobbels | | Assessing COPD patients' experiences of physical activity: From qualitative interviews to item generation |  |
| 32 | 2010 | Matheson | | COPD Patients derived benefits from attending PR: ‘This has given me my life back’ |  |
| 33 | 2022 | Marklund | | The importance of feeling in control - people with COPD's experiences regarding maintaining or increasing physical activity when using an eHealth tool. A grounded theory analysis |  |
| 34 | 2022 | Turner | | Photovoice exploration of physical activity norms and values among rural and remote pulmonary rehabilitation participants in British Columbia, Canada |  |
| 35 | 2023 | Padhye | | Perspectives of Patients With Chronic Respiratory Diseases and Medical Professionals on Pulmonary Rehabilitation in Pune, India: Qualitative Analysis | Study participant incompatibility |
| 36 | 2023 | Sami | | Barriers to adherence to home-based pulmonary rehabilitation among patients with chronic obstructive pulmonary disease in Iran: a descriptive qualitative study |  |
| 37 | 2020 | Rayce | | Telemediated Training in the Home as a Part of the Everyday Life and Practice With Very Severe Chronic Obstructive Pulmonary Disease |  |
| 38 | 2020 | Miranda | | Exploring transitions in care from pulmonary rehabilitation to home for persons with chronic obstructive pulmonary disease: A descriptive qualitative study |  |
| 39 | 2020 | Philip | | Dance for people with chronic respiratory disease: a qualitative study |  |
| 40 | 2019 | Nakken | | Identifying causes of perceptual differences in problematic activities of daily life between patients with COPD and proxies: A qualitative study |  |
| 41 | 2018 | Jones | | Does pulmonary rehabilitation alter patients' experiences of living with chronic respiratory disease? A qualitative study |  |
| 42 | 2019 | Jakob | | Early supported discharge with pulmonary rehabilitation following exacerbations of COPD | Inconsistency in type of study |
| 43 | 2017 | Lindenmeyer | | How Do People With COPD Value Different Activities? An Adapted Meta-Ethnography of Qualitative Research |  |
| 44 | 2010 | Vieira | | Home-based pulmonary rehabilitation in chronic obstructive pulmonary disease: The patient experience |  |
| 45 | 2023 | Midthun | | Understanding the Patient Experience of Home-Based Pulmonary Rehabilitation with Health Coaching for COPD: A Qualitative Interview Study | Inconsistency of study phenomena |
| 46 | 2023 | Gabriel | | Patient Perceptions of a Virtual Reality-Based System for Pulmonary Rehabilitation: A Qualitative Analysis. |  |
| 47 | 2023 | Papp | | Experiences of physical activity and exercise among women with obstructive pulmonary disease |  |
| 48 | 2023 | Riquier | | Capitalization of experience of physical activity resumption programs: Lessons learned for adherence and post-program referral |  |
| 49 | 2022 | Grigoletto | | Satisfaction and Adherence of COPD Patients to a Conventional Training Associated with Functional Exercises and to a Conventional Training Isolated: a Qualiquantitative Study |  |
| 50 | 2022 | Skibdal | | Listen to Me! – A Mixed-Methods Study of Thoughts and Attitudes Towards Participation in Pulmonary Telerehabilitation Among People with Severe and Very Severe COPD Who Declined Participation in Pulmonary Rehabilitation |  |
| 51 | 2022 | Simony | | Experiences in responders and non-responders to pulmonary rehabilitation among people with chronic obstructive pulmonary disease: a clinical study with convergent mixed analysis |  |
| 52 | 2021 | Scheermesser | | The influence of pulmonary rehabilitation and counselling on perceptions of physical activity in individuals with copd – a qualitative study |  |
| 53 | 2021 | Gilliam | | The impact of Tai Chi and mind-body breathing in COPD: Insights from a qualitative sub-study of a randomized controlled trial |  |
| 54 | 2021 | Baxter | | Perceptions and experiences of a manual therapy trial: a qualitative study of people with moderate to severe COPD |  |
| 55 | 2021 | 洪涵涵 | | COPD病人肺康复运动训练的质性研究 |  |
| 56 | 2020 | Meshe | | Participants' experiences of the benefits, barriers and facilitators of attending a community-based exercise programme for people with chronic obstructive pulmonary disease |  |
| 57 | 2020 | Brighton | | Experiences of Pulmonary Rehabilitation in People Living with Chronic Obstructive Pulmonary Disease and Frailty. A Qualitative Interview Study |  |
| 58 | 2020 | Spitzer | | "You Leave There Feeling Part of Something": A Qualitative Study of Hospitalized COPD Patients' Perceptions of Pulmonary Rehabilitation |  |
| 59 | 2019 | O'Connor | | Is inspiratory muscle training (IMT) an acceptable treatment option for people with chronic obstructive pulmonary disease (COPD) who have declined pulmonary rehabilitation (PR) and can IMT enhance PR uptake? A single-group prepost feasibility study in a home-based setting |  |
| 60 | 2018 | Papp | | Experiences of hatha yogic exercises among patients with obstructive pulmonary diseases: A qualitative study |  |
| 61 | 2017 | Genoe | | How Lived Body and Lived Space Intersect to Influence the Experience of Chronic Obstructive Pulmonary Disease |  |
| 62 | 2016 | 刘泓 | | 老年COPD稳定期患者家庭肺康复自我体验的质性研究 |  |
| 63 | 2016 | Levack | | Whakawhanaungatanga: the importance of culturally meaningful connections to improve uptake of pulmonary rehabilitation by Māori with COPD - a qualitative study |  |
| 64 | 2016 | Hoaas | | Adherence and factors affecting satisfaction in long-term telerehabilitation for patients with chronic obstructive pulmonary disease: a mixed methods study |  |
| 65 | 2015 | Nordvall | | Enhanced information regarding exercise training as treatment is needed. An interview study in patients with chronic obstructive pulmonary disease |  |
| 66 | 2014 | Lewis | | Uncertainty prior to pulmonary rehabilitation in primary care: A phenomenological qualitative study in patients with chronic obstructive pulmonary disease |  |
| 67 | 2014 | Desveaux | | Participant experiences of a community-based maintenance program post-pulmonary rehabilitation |  |
| 68 | 2013 | Tang | | Patients admitted with an acute exacerbation of chronic obstructive pulmonary disease had positive experiences exercising from the beginning of their hospital stay: a qualitative analysis |  |
| 69 | 2013 | Marques | | Comprehensive ICF Core Set for Obstructive Pulmonary Diseases: Validation of the Activities and Participation component through the patient's perspective |  |
| 70 | 2013 | Wang | | An exploration of beliefs regarding exercise among Taiwanese patients with chronic obstructive pulmonary disease |  |
| 71 | 2012 | Hellem | | Exercise maintenance: COPD patients' perception and perspectives on elements of success in sustaining long-term exercise |  |
| 72 | 2012 | Moore | | Acceptability and feasibility of pulmonary rehabilitation for COPD: a community qualitative study |  |
| 73 | 2011 | Williams | | The importance of movement for people living with chronic obstructive pulmonary disease |  |
| 74 | 2011 | Keating | | Lack of perceived benefit and inadequate transport influence uptake and completion of pulmonary rehabilitation in people with chronic obstructive pulmonary disease: a qualitative study |  |
| 75 | 2010 | Hellem | | Facilitators and barriers to exercise maintenance in chronic obstructive pulmonary disease: Patient views |  |
| 76 | 2008 | Harris | | Improving the uptake of pulmonary rehabilitation in patients with COPD: qualitative study of experiences and attitudes |  |
| 77 | 2007 | O'Shea | | Qualitative outcomes of progressive resistance exercise for people with COPD. |  |
| 78 | 2007 | Fischer | | Participation and drop-out in pulmonary rehabilitation: a qualitative analysis of the patient's perspective |  |
| 79 | 2007 | Melhuish-Williams | | A breath of fresh air : the experience of activity in Chronic Obstructive Pulmonary Disease |  |
| 80 | 1994 | McBride | | Patients with chronic obstructive pulmonary disease: their beliefs about measures that increase activity tolerance |  |
| 81 | 2021 | de Carvalho | | Quali-quantitative analysis of adherence and perceived satisfaction of individuals with COPD after high-intensity training on land and in water: additional analysis from a randomized clinical trial | Qualitative data could not be separated |
| 82 | 2018 | Loeckx | | Smartphone-based physical activity telecoaching in chronic obstructive pulmonary disease: mixed-methods study on patient experiences and lessons for implementation |  |
| 83 | 2020 | Gilliam | | Impact of tai chi and mind-body breathing in chronic obstructive pulmonary disease: insights from a qualitative substudy of a randomized controlled trial | Duplicate publications |
| 84 | 2020 | Kaptain | | Everyday Life Activities of People with Chronic Obstructive Pulmonary Disease: Performance, Management and Everyday Technology Use |  |

**Appendix 5: Findings and illustrations**

| **Illustrations** | **Findings** | **Categories** | **Synthesized findings** |
| --- | --- | --- | --- |
| Levels of activity go up and down, my lungs do not stay the same all the time…because in three weeks three hours, your breathing could be completely different. [Hogg et al., 2012] | Variability and unpredictability of symptoms (unequivocal) | Facing with fluctuations in symptoms and post-activity discomfort | Confronting symptom management and psychological challenges |
| So, when I feel bad, I can't move myself, I can't take a shower, I can't get dressed. I can't do anything. It is cruel...[Kaptain et al., 2022] | Affected by acute deterioration of the condition (unequivocal) |  |  |
| I do the kitchen, clean down the woodwork and when you bend down and get up you’re short of breath.[Kosteli et al., 2017] | Experienced difficulty breathing after activities (unequivocal) |  |  |
| When I get out, I am more dead than alive. I get out from the shower and go sit on the toilet, I put a blanket or a towel on the floor and dry my feet there. Then I sit there and dry. There is heat out there. Well, about one and a half, I don’t know how long it takes. I guess it takes a half hour or three quarters before I have my clothes on.[Kaptain et al., 2022] | Experienced fatigue after activities (unequivocal) |  |  |
| I’m not confident of going out. What if I get breathless when I’m outside?[Chang et al., 2022]  What I need is to rest, I used to not pay much attention to it all back and forth into the hospital many times, the mood is very bad, and when I get out of the hospital, I don't want to do anything, or afraid to do it![Luo et al., 2020] | Low self-efficacy in community activities (unequivocal) | Encountering psychological and emotional challenges |  |
| When I first got the pains when I went up steps or walking up a hill, it used to actually frighten me a bit, I used to think oh god this is my heart or you know.[Kosteli et al., 2017] | Concerns about breathing difficulties and pain (unequivocal) |  |  |
| I won’t leave my home, even if someone is accompanying me. I am afraid of causing trouble for my family.[Jeng et al., 2002] | Fear of becoming an additional burden to family members (unequivocal) |  |  |
| …I did not [continue] because [I] just sat down and lifted my leg…I find that it doesn’t help.[Chang et al., 2022] | Poor adherence to exercise recommendations (unequivocal) | Experiencing non-adherence and individual differences | Lacking compliance, behavior change capacity, and autonomy in decision-making |
| A lot of [the exercises] were very interesting but personally I had such strong legs they weren’t doing me much good at the time. But you have to realize I’ve been a skier all my life…I worked outside…it’s just in the past, oh, I don’t know, 6 to 8 months I guess, I started losing muscle. [Chauvin et al., 2022] | Varying levels of personal physical health (unequivocal) |  |  |
| I suppose the other thing is my age. I’m 71…my age is such that it’s a limit, I guess, to what I could do and the way that anything that’s required, it’s difficult.[Thorpe et al., 2014] | Aging limits on activity capability (unequivocal) | Lacking the ability to translate intentions into actions |  |
| I mean, I would love to do (Activities of daily living [ADL] such as housework), but I’ve got to carry my battery with the oxygen, because the government doesn’t give you that-it’s a beautiful battery you can carry…but I have to buy it myself, it would cost me $6,000 and I can’t afford it.[Thorpe et al., 2014] | Economic burden of carrying oxygen equipment (unequivocal) |  |  |
| If it gets too cold, I won’t go out at all. Or if it’s too humid in the warmer weather.[Thorpe et al., 2014] | Seasonal weather patterns reduce outdoor activity ability (equivocal) |  |  |
| My husband takes me everywhere and he does everything, he won’t let me do anything though… It is a problem when somebody is over-protective.[Kosteli et al., 2017]  I'm not even asked to do simple chores much anymore. I used to be the one going downstairs every day to buy groceries, but now it's much less frequent.[Luo et al., 2020] | Overprotection by family members impedes patient activity (unequivocal) | Experiencing an imbalance between social support and autonomous decision-making |  |
| …I was offered help when taking a shower, but I said no to that. At the hospital, I got help, and I liked that, but I cannot get up in the morning and just say “now, I need to shower”, just because someone is coming to help. I want to decide by myself when I would like to shower.[Kaptain et al., 2022] | Resistance to dependency (unequivocal) |  |  |
| A: Our goals are not hard. Just to be well.  B: Yeah. Enjoy life.  C: And be able to manage in the house and go out.[Michalovic, 2021] | Alignment of activity projects with personal goals (unequivocal) | Striving for personal goals and psychological rewards | Stimulating motivations for engaging in activities |
| ... and doing the walking every day or attempting to walk every day is really good for the chronic fatigue also and for my osteoporosis as well as my emphysema so it has been great and I have noticed lots of small changes in my walking[Lahham et al., 2018]. | Achievement of personal goals (equivocal) |  |  |
| I’m motivated by the social side, the people I meet with or play. I’ll play any sport just for the social side of it; yeah I do enjoy that, that’s a motivation.[Kosteli et al., 2017]  I found that it was lovely to just walk past gardens and, you know, just see what is going on around and even people will speak with you, you know. It just cheers you up for the day and you look at life differently[Lahham et al., 2018]. | Social interaction through activities (unequivocal) |  |  |
| My sons and daughters are very dutiful, they take us old folks out of the province every year, and I need to prepare for the trip to avoid having to walk a few steps.[Luo et al., 2020] | Preparation for upcoming events (unequivocal) |  |  |
| I will try to [go to the market] anyway. I don't know how long I can walk, but I'll try[Leidy et al., 1996]. | Seeking satisfaction through activities (unequivocal) |  |  |
| Motivation to feel stronger. I know that sounds like it was idealistic answers. But that’s the way I felt. Some days I didn’t feel like doing it, of course… you wake up and you’re, “really, do I have to?” But then the alternative for me is…to go backwards with the COPD.[Chauvin et al., 2022] | Self-motivation through commitment, determination, and guilt (unequivocal) | Self-motivating and receiving intuitive feedback |  |
| I wear my [exercise] bracelet to see how active I am for the day, and if I don't reach my goal, I'll stick with it, and I'm walking more and more now.[Luo et al., 2020] | Visual motivation through testing and feedback (unequivocal) |  |  |
| Well, I can walk a bit further now and I am thinking to myself ‘Breathe! Breathe! You can do it!’ so I talk to myself down the street. I am going a bit better with my breathing[Lahham et al., 2018]. | Building confidence in activity through controlled breathing (unequivocal) | Coordinating activities and managing respiratory difficulties | Managing activity plans, respiratory aspects during activities, and activity safety |
| I do a bit of woodwork here at home, just sort of hobby stuff. I’ve got a few things that I just mess around with, and I have to be really careful with anything that produces dust or anything like that, because obviously that gives me a problem with breathing, or can do.[Thorpe et al., 2014] | Awareness of pollen and dust in activity environments (unequivocal) |  |  |
| You can't run up the stairs anymore. You have to hold yourself back and take the steps in a speed that allows you to maintain your breathing without getting out of breath partway up. I came by [subway]. And the escalator was broken at the [subway] station, so I had to walk up several flights of stairs. I did it all at once, but I did it slowly. You let the other people go. Don't try to keep up with anybody, hang onto the rail, and take it easy. And sure, you're going to be puffing a little more by the time you get to the top, but you're not going to be totally out of breath. If you go too fast, you have to stop and recover.[Michalovic, 2021] | Adapting to personal activity capabilities (unequivocal) |  |  |
| When I breathe heavily, I will take a bath slowly…I will sit down to rest when I feel myself breathing heavily…Compared to before, I need more time to take a bath. I sit in the bath room and take a shower on a stool. I use a towel with some soap on it to rub my back. And it is easier for me.[Jeng et al., 2002] | Increasing rest time and simplifying activities (unequivocal) |  |  |
| I have the idea in my mind if it’s less than two miles or up to two miles I will walk rather than take the car or the bike.[Kosteli et al., 2017]  It means everything to stay active. I also sing in the church choir, which means that you have to use your lungs differently, and your breathing is also important…I do not have the same energy as I used to. There are some things I really try to hold on to, and that is exercise and the leisure activities I attend-they are important.[Kaptain et al., 2022] | Incorporating activities into daily life and setting goals (unequivocal) | Adapting daily activity schedules flexibly |  |
| Yes! I get a little bit puffy in the mornings, so I do them in the afternoons[Lahham et al., 2018].  I have to get everything done in the morning...because in the afternoon, I'm done [exhausted][Leidy et al., 1996]. | Problem-solving and activity planning (unequivocal) |  |  |
| During the program, I had a cold and we put [the program] off…put it off for a couple of weeks…until I kind of could breathe better…So that was good for me.[Chauvin et al., 2022] | Adjusting based on health conditions (unequivocal) |  |  |
| Don’t grab any thing too heavy, try to pick up only some light weight things. Please do not exercise strenuously, just do it naturally, walk slowly, don’t force yourself. Do what ever you can do. If you do not feel right about doing it, then just quit. Don’t do it reluctantly.[Jeng et al., 2002] | Proceeding within one's capabilities (unequivocal) |  |  |
| During the first few days when I had just come back from the hospital, I gradually resumed my morning activities. I divided them into two to three sections to allow myself to recover to the level that I used to have. Do it slowly. Rest when tired. Don’t walk for too long or strenuously, otherwise, I might fall down.[Jeng et al., 2002] | Gradual progression (unequivocal) | Strengthening self-management of activity safety |  |
| I am safe at home; you see I have furniture I can hold on to every where in my house. I won’t fall down. I even have a chair in my bathroom to support me from falling there. So I am absolutely safe at home.[Jeng et al., 2002] | Selecting appropriate assistive tools (unequivocal) |  |  |
| When it gets time for dinner, I have this prepared food. My children made it for me. I just reach down the freezer-what would you like?…My daughter-in-law always bakes for me.[Kaptain et al., 2022] | Special care from family members (unequivocal) | Providing personalized family support | Optimizing family, peer, and medical support systems |
| I also think that support from family, ie, your partner, husband, or wife, is pretty important on a day-to day routine. My wife might say to me, nice day out, so you fancy taking the dog for a walk in the park…there’s a backing there.[Kosteli et al., 2017] | Understanding and encouragement from spouse (unequivocal) |  |  |
| I think it can help you to realize what you can or cannot do. Because if you see others doing a little more than you, and they have the same condition, you think then, "Well, if they can do it, possibly I can do it." And then you can try a little more and push yourself a little more. Because the tendency always is to fall back, that's the easiest. You get a little out of breath, so you stop. But if you find that somebody else is doing the same thing just a little more than you, then possibly you can do that too. So, it's a little peer support and peer pressure that can help[Michalovic, 2021]. | Peer support enhances activity capabilities (unequivocal) | Enhancing peer support networks |  |
| We are around 8 people at the group [COPD choir]. We have been together for a few years now, and we sing two hours a week. Well, we don’t, we practice breathing techniques and it is so good. We talk to each other…both about techniques, and about what we do and what has been good to do.[Kaptain et al., 2022] | Establishing connections within peer groups (unequivocal) |  |  |
| It would be great if there were places where patients like us could move around together and also supervise us.[Luo et al., 2020] | Lack of community activity environments (unequivocal) |  |  |
| [In hospital] they just ask me to walk around slowly…She just held me and walked around then asked me to rest.[Chang et al., 2022] | Disappointing experience with inpatient physical therapy (equivocal) | Expanding the content and models of medical guidance |  |
| The doctors talked to me before I was discharged from the hospital, I can't be afraid to move because of this disease, they instructed me on what to do when I have trouble breathing, I can handle it calmly now, and I'm not so scared anymore when I have my medication with me.[Luo et al., 2020] | Encouragement and professional guidance from attending physicians (unequivocal) |  |  |
| I loved when [the physiotherapist] came because she was so appreciative of what I was doing. So she made me feel good…Because she could see the improvement…you had this person…keeping you going…And then, you know, I’d get a pat on the back because I was improving, which is nice.[Chauvin et al., 2022] | Home visits and follow-ups by physical therapists (unequivocal) |  |  |
| And you can think of in the future of videos that people could have on their iPad and iPhone, just to help us[Michalovic, 2021]. | Participation in activity programs via online platforms (unequivocal) |  |  |
